# TimTrack: A drift-free algorithm for estimating geometric muscle features from ultrasound images

**DOI:** 10.1371/journal.pone.0265752

**Published:** 2022-03-24

**Authors:** Tim J. van der Zee, Arthur D. Kuo

**Affiliations:** Biomedical Engineering Graduate Program, Faculty of Kinesiology, University of Calgary, Calgary, Alberta, Canada; Johns Hopkins University, UNITED STATES

## Abstract

Ultrasound imaging is valuable for non-invasively estimating fascicle lengths and other features of pennate muscle, especially when performed computationally. Effective analysis techniques to date typically use optic flow to track displacements from image sequences, but are sensitive to integration drift for longer sequences. We here present an alternative algorithm that objectively estimates geometric features of pennate muscle from ultrasound images, without drift sensitivity. The algorithm identifies aponeuroses and estimates fascicle angles to derive fascicle lengths. Length estimates of human vastus lateralis and gastrocnemius fascicles in healthy subjects (N = 9 and N = 17 respectively) compared well (overall root-mean-square difference, RMSD = 0.52 cm) to manual estimates by independent observers (n = 3), with overall coefficient of multiple correlation (CMC) of 0.98. Our tests yielded accuracy (CMC, RMSD) and processing speed similar to or exceeding that of state-of-the-art algorithms. The algorithm requires minimal manual intervention and can optionally extrapolate fascicle lengths that extend beyond the image frame. It thus facilitates automated analysis of ultrasound images without drift.

## Introduction

Ultrasonography, or ultrasound, can be used to non-invasively estimate features of pennate muscle geometry, including muscle thickness [e.g. 1], pennation angle [e.g. 2] and fascicle length [e.g. 3]. These features are of particular interest in biomechanics, because they influence both mechanical [e.g. 4–6] and energetic aspects of muscle force production [e.g. 7–9]. Whereas ultrasound analysis was originally performed manually, (semi-)automated methods have recently become more prevalent [for a recent review, see 10]. Unfortunately, existing (semi-)automated methods that estimate fascicle length can be sensitive to drift if using optic flow, and some require considerable user interaction. It is thus helpful to devise an algorithm that is less sensitive to drift, more automated, and freely available.

One of the challenges with automated ultrasound analysis is image speckle, a type of interference that degrades image quality and hampers tracking of single fascicles [10]. Sensitivity to image speckle can be reduced by using optic flow, which is one of the primary (semi-)automated methods. Optic flow uses the image velocity field to indicate bulk changes that are relatively insensitive to speckle, and thus estimates fascicle length changes between succeeding image frames [11–13]. One shortcoming is that accuracy requires succeeding images to be similar, such that faster muscle contraction requires high image capture rates [10]. Another drawback is that optic flow techniques accumulate the frame-by-frame changes over time, which also accumulates errors over long sequences, referred to as integration drift. Optic flow algorithms are therefore also history dependent, and usually need an initial, manually-tracked image that may introduce subjectivity from human operators. A final, manually-tracked image at the end of an image sequence may also be used to reduce drift, but again with some subjectivity. Optic flow techniques are therefore most effective for relatively short image sequences captured at high rates, and typically require significant user intervention.

Another approach is automated feature detection within a single image, rather than optic flow across multiple images. Detected features may include muscle thickness [14, 15], fascicle orientation [16–19, 15], pennation angle [15, 20] and fascicle length [15, 21]. To reduce influence of speckle, feature detection algorithms typically rely on image filtering procedures. Filtered images may be used to identify individual fascicle objects [18] and features such as fascicle orientation, or to obtain aggregate feature estimates using line detection procedures [15, 19]. Although such detection is history independent, most feature detection algorithms still rely on some degree of manual intervention, for example to detect the aponeuroses [e.g. 18, 19], which helps to define fascicle lengths. However, others have used feature detection techniques to also automate aponeurosis detection [15, 21]. It is thus possible to automate most detection steps, yielding a more objective, repeatable, and convenient means of estimating muscle fascicle features without drift.

Here we present an ultrasound algorithm called TimTrack that uses a combination of existing feature detection techniques and new procedures to automatically estimate geometric features of pennate muscle without drift. Existing feature detection techniques employed by TimTrack include vessel enhancement image filtering to highlight aponeuroses and fascicles, and Hough transform to estimate overall fascicle- and aponeurosis orientation. New procedures include detecting the inner edges of aponeuroses, optional object detection and polynomial fitting to identify curvilinear aponeuroses, image rotation to reduce bias of Hough transform, and weighted-median fascicle angle estimation for better robustness against outliers. These techniques and procedures are selected based on both accuracy- and processing speed considerations. We qualitatively compare TimTrack’s performance against three other freely-available, state-of-the-art algorithms, including the UltraTrack and Point-tracking optic flow algorithms [12, 13] and the SMA feature detection algorithm [15]. We here show that TimTrack’s estimates are comparable to those from manual observers, with similar accuracy as the other three algorithms, at faster processing speed than the SMA feature detection algorithm, and without the drift sensitivity of optic flow algorithms.

## Methods

The developed TimTrack algorithm estimates geometric muscle features from ultrasound images using several feature detection techniques. The algorithm employs a filter to highlight line-like structures such as aponeuroses and fascicles in the images. After automatically detecting the superficial- and deep aponeuroses in the image, it uses a technique called the Hough transform to estimate the overall fascicle orientation. These data, together with geometric calculations, yield estimates of muscle thickness, pennation angle, and muscle fascicle length. We tested the algorithm on ultrasound images obtained from vastus lateralis and gastrocnemius muscle in healthy human subjects and compared its estimates to manual estimates from independent observers (N = 3).

### Algorithm

**Fig 1:**
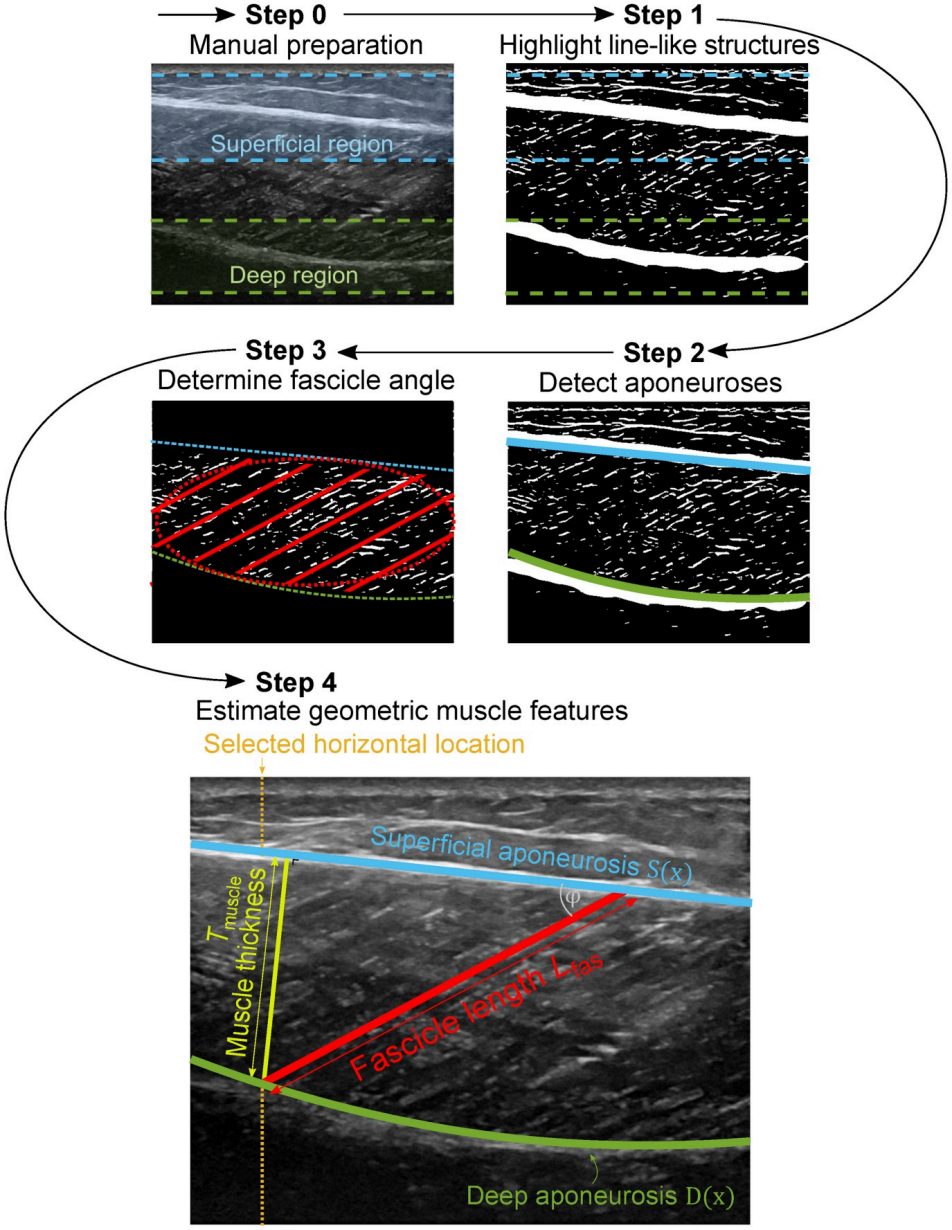
Summary of steps in processing of ultrasound images. Raw images undergo manual preparation to select regions (step 0), and image filtering to highlight line-like structures (step 1). Aponeuroses are detected from the filtered images using either object- or line detection procedures (step 2). An ellipsoid region between detected aponeuroses is used to determine fascicle angle with a line detection procedure (step 3). Geometric muscle features are estimated from detected aponeuroses and determined fascicle angle (step 4). Pennation angle *φ* is defined as the difference between fascicle angle and superficial aponeurosis angle. Muscle thickness *T*_muscle_ is defined as the perpendicular distance from the deep aponeurosis to the detected superficial aponeurosis. Fascicle length *L*_fas_ is calculated from pennation angle *φ* and muscle thickness *T*_muscle_ using trigonometry. Muscle thickness *T*_muscle_ and therefore fascicle length *L*_fas_ can be evaluated at any horizontal location selected by the user (dotted yellow line).

There are four main steps to the algorithm (see Fig 1), and two additional, optional steps. Prior to its application, there is a manual (0.) preparation step in which the user specifies two image regions of interest where the aponeuroses are to be detected. The subsequent steps are automated: (1.) Highlight line-like structures for fascicles (thin lines) and aponeuroses (thick lines). (2.) Detect aponeuroses and their inner edges. (3.) Determine fascicle angles. (4.) Estimate geometric muscle features, including muscle thickness *T*_muscle_, pennation angle *φ* and fascicle length *L*_*fas*_. In addition, two optional steps may be performed depending on the application: (5.) Extrapolate geometry beyond the image frame for longer fascicles. (6.) Time-interpolate aponeuroses for image sequences with missing data (e.g., from occlusion). These steps are explained in greater detail next.

#### Preparation

Ultrasound images should be cropped to exclude extraneous borders and labels, which are added by some commercial systems. Within the actual ultrasound data image, the algorithm assumes that fascicles are oriented within the first quadrant of the x-y plane, so that pennation angle is measured as counter-clockwise with respect to the horizontal. (If necessary, the image may be flipped or reoriented accordingly.) Before applying the algorithm, the user selects two regions of interest where the superficial and deep aponeuroses are to be detected. This assumes an image where the superficial aponeurosis is near the top, and the deep aponeurosis is near the bottom. The regions are specified by a relative depth range for each aponeurosis (*D*_superficial_ and *d*_deep_, see S1 Appendix). In practice, these regions need normally be specified only once for an entire data set, if images are acquired in a consistent procedure.

#### Highlight line-like structures

In this first step, line-like structures in the ultrasound image are highlighted to facilitate both muscle fascicle- and aponeurosis detection. This highlighting procedure incorporates the Frangi-type vessel enhancement filter [22], which was originally developed for enhancing blood vessels in imaging and has later been applied to enhancing line-like muscle fascicles [17, 19] and aponeuroses [14]. We adapted an open-source implementation of this filter [23] to reduce edge effects, following a user suggestion to “adding the ’replicate’ argument to the imfilter call in Hessian2D.m” (user Phillip, Matlab Central). The adapted filter is applied separately for fascicles and aponeuroses using different line thickness settings (e.g. thicker lines for aponeuroses, thinner lines for muscle fascicles), to yield two filtered images.

**Fig 2:**
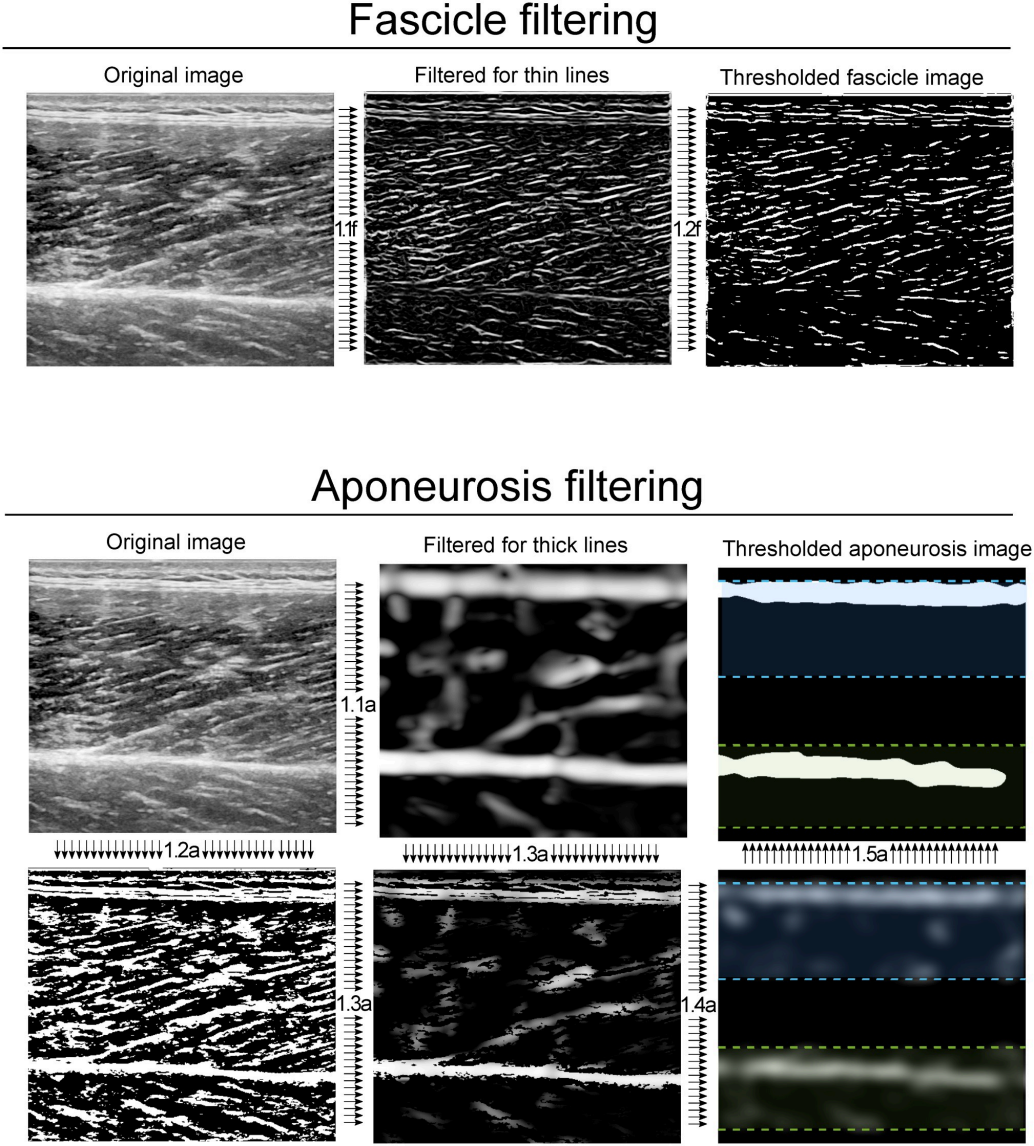
Image filtering steps for highlighting line-like structures. Fascicle filtering includes highlighting thin lines using a vessel enhancement filter (step 1.1f) and subsequent thresholding (step 1.2f). Aponeurosis filtering includes highlighting thick lines (step 1.1a), masking with a thresholded version of the original image (step 1.2a and step 1.3a), Gaussian smoothing (step 1.4a) and thresholding (step 1.5a). The results of the fascicle and aponeurosis filtering process are inputs for the fascicle angle determination (step 3) and aponeurosis detection respectively (step 2).

*Fascicle filtering*. The vessel enhancement filter is applied with fascicle thickness parameter *σ*_fas_ (see S1 Appendix), relating to the (average) fascicle thickness in terms of image pixels. After the vessel enhancement filter is applied (step 1.1f, Fig 2), the filtered fascicle image is thresholded (step 1.2f, Fig 2, *T*_fas_, see S1 Appendix).

*Aponeurosis filtering*. The vessel enhancement filter is applied with aponeurosis thickness parameter *σ*_apo_ (see S1 Appendix), relating to the (average) aponeurosis thickness in terms of pixels. After the vessel enhancement filter is applied (step 1.1a, Fig 2), the aponeurosis image is masked (step 1.3a, Fig 2). The mask is created by thresholding the original image (step 1.2a, Fig 2, *T*_apo_, see S1 Appendix), and applied by multiplying against the filtered aponeurosis image, to reduce boundary effects of the vessel enhancement filter. Next, a second mask is applied to retain only the user-defined depth region for each aponeurosis and the remaining white pixels in each depth region (i.e. superficial and deep) are smoothed using a 2D Gaussian kernel (*imgaussfilt*, MATLAB, step 1.4a, Fig 2) with a standard deviation of half an aponeurosis thickness (i.e. $\frac{\sigma_{apo}}{2}$, see S1 Appendix), and thresholded (step 1.5a, Fig 2). We recommend using the aponeurosis filter when using the ‘object detection method’ for aponeurosis detection; the filter may be omitted when using the ‘Hough transform method’ for aponeurosis detection (see “Detect aponeuroses and their inner edges”).

#### Detect aponeuroses and their inner edges

In the second step, the superficial and deep aponeuroses are detected in the aponeurosis image. TimTrack’s default method for aponeurosis detection is the Hough transform, a feature detection technique commonly used for detecting straight lines in images [24]. We found this method to work well when aponeuroses resemble straight lines, even when they are not clearly visible. Aponeurosis filtering may be omitted when using the Hough transform, speeding up the overall image analysis by about 40%. If aponeuroses do not resemble straight lines but are curved and/or blob-like, the straight-line assumption in the Hough transform may be invalid. In this case, the user may choose to perform the alternative object detection method instead.

*Step 2*.*1*: *Hough transform aponeurosis detection method (default)*. The Hough transform aponeurosis detection method detects aponeuroses in the thresholded aponeurosis image by assuming straight lines through the aponeuroses to encounter more white pixels than any other straight line. First, the Hough transform is applied to each depth region (*hough*, MATLAB), resulting in two 2D accumulator matrices. Each 2D accumulator matrix represents frequency counts of lines that are parameterized by angle *θ* and distance *ρ*, using a predefined angle resolution (*θ*_apo,res_, see S1 Appendix) and distance resolution of 1 pixel. The angle-distance combination with the highest accumulator count corresponds to the most ‘dominant’ line in the image. A shortcoming of this procedure is that it favours horizontal, vertical, and diagonal lines disproportionately, owing to pixels being square. TimTrack corrects for this bias by also applying the Hough transform to a rotated version of the image (*imrotate*, MATLAB), and replacing the horizontal, vertical, and diagonal accumulator values in the original matrix with the ones from the rotated image. Next, angle *θ* and distance *ρ* with the maximum accumulator value are determined for each depth region, and the corresponding lines are evaluated at a predefined number of equidistantly spaced horizontal locations (*n*_apox_, see S1 Appendix) across the image width, with specified margin from the image boundary (*x*_margin_, see S1 Appendix). The latter allows for cropping from each side to reduce edge effects. Whereas the evaluated points are typically located in the middle of the aponeuroses, the inner edge is a more relevant geometric feature because that is where the fascicles attach. If the evaluated point corresponds to a white pixel, it is therefore traced vertically towards the fascicles until reaching the inner edge (defined as the depth location of the last observed white pixel before encountering a black pixel).

*Step 2*.*2*: *Object detection aponeurosis method (alternative)*. As an alternative to the Hough transform method, TimTrack’s object detection method can detect aponeuroses without assuming straight lines. This method requires images of relatively low speckle and high resolution and should be used in combination with aponeurosis filtering (see step 1). The object detection method seeks to find the collection of continuously adjacent white-pixels (together called an ‘object’) in the thresholded aponeurosis image that belongs to each aponeurosis. First, the two longest objects in each depth region (i.e., superficial and deep) of the image are selected. If one is clearly longer and the length ratio between the smallest and longest of these two objects is less than a pre-set parameter value (*L*_ratio,max_, see S1 Appendix), the longest object is designated as the aponeurosis object (*bwpropfilt*, MATLAB, using the *Major Axis Length property*). If the two longest objects have similar length and the length ratio is larger than the parameter value, the objects are used to mask the grayscale (i.e. non-thresholded) image. The object resulting in the masked grayscale image with the highest mean pixel intensity is coined the aponeurosis object (*bwpropfilt*, MATLAB, using the *Mean Intensity property*). Next, the inner edges of the superficial and deep aponeurosis objects are determined, and used to define sampled aponeurosis points. The inner edges are defined as the white pixels on the aponeurosis objects closest to the interior of the image. The edges are first trimmed to compensate for the width added by the 2D Gaussian kernel used in step 1.4a. This is done by vertically shifting both aponeurosis objects outward (i.e. away from the fascicles) over a distance of half the standard deviation of the kernel (i.e. $\frac{\sigma_{apo}}{4}$). The superficial and deep aponeurosis points are defined as these corrected inner edges, evaluated at a predefined number of equidistantly spaced horizontal locations (*n*_apox_, see S1 Appendix) across the image width, with specified margin from the image boundary (*x*_margin_, see S1 Appendix).

*Step 2*.*3*: *Fitting aponeurosis lines*. Both aponeurosis detection methods result in a collection of aponeurosis points, located on the inner edges of the aponeuroses. Next, superficial and deep aponeurosis lines (*y* = *S*(*x*) and *y* = *D*(*x*) respectively, solid blue and green lines in Fig 1) are obtained from the aponeurosis points, through fitting polynomials of specified order (*o*_deep_ and *o*_super_, see S1 Appendix) using constrained (least-squares) optimization (*fmincon*, MATLAB). Constraints are placed on the maximal value of the lines’ derivative, derived from the range of eligible aponeurosis angles (*β*_range_ and *γ*_range_, see S1 Appendix).

#### Determine fascicle angles

In the third step, fascicle angle is determined by applying the Hough transform to the filtered fascicle image. First, the fascicle region-of-interest is defined as an elliptic area, spanning between the detected aponeuroses (see step 2) and across the entire image width (dotted red line, Fig 1). The user can choose between calculating the elliptic area once per image sequence to speed up the analysis, or once per image for better accuracy. The Hough transform is applied to the ellipsoid fascicle image (*hough*, MATLAB), using a predefined angle resolution (*θ*_fas,res_, see S1 Appendix), angle range (*θ*_fas,range_, see S1 Appendix) and distance resolution of 1 pixel. To reduce bias towards horizontal, vertical and diagonal lines, we also apply the Hough transform to a rotated version of the image, and replace the original accumulator values of these lines with corresponding values of the rotated transform. Next, the 2D accumulator matrix is corrected for the effect of angle *θ* on the relative ellipse radius. The peaks in the corrected 2D accumulator matrix are determined (*houghpeaks*, MATLAB) and the angles *θ* belonging to the highest peaks are selected, using a pre-set number of included peaks (*K*, see S1 Appendix). Fascicle angle *α* is defined as the weighted median of the selected angles, with each angle weighted by its corresponding accumulator value. We chose to use the median instead of the mean for better robustness to outliers.

#### Calculate muscle thickness, pennation angle and fascicle length

In the fourth step, pennation angle *φ* and muscle fascicle length *L*_fas_ are determined from aponeurosis fits and fascicle angle *α*. Pennation angle *φ* is defined as the difference between superficial aponeurosis angle *β* and the fascicle angle *α*,

φ=α-β

Superficial aponeurosis angle *β* is defined as the angle of the superficial aponeurosis line *S*(*x*) at the right image border. Muscle thickness *T*_muscle_ is defined as the perpendicular distance from the (fitted) deep aponeurosis to the (fitted) superficial aponeurosis, and can be evaluated at any horizontal location *x* (see Fig 1):

$$T_{muscle}(x,\beta )=|S\left(x\right)-D\left(x\right)|∙cos(\beta )$$

Fascicle length *L*_fas_ is calculated from pennation angle *φ* and muscle thickness *T*_muscle_ using trigonometry (see Fig 1),

$$L_{fas}\left(x,\beta ,\varphi \right)=T_{muscle}\left(x,\beta \right)∙{sin(\varphi )}^{-1}$$

Note that if *L*_fas_ extends beyond the visible ultrasound image, the fascicle and aponeurosis will be extrapolated. This assumes that the fascicle and aponeurosis geometry apply throughout the extrapolation region, which the user may assess by imaging that region (e.g., panoramic images or dual probe in some systems). To alert the user of extrapolation, TimTrack shows an estimate of the extrapolated fraction of *L*_fas_ and warns when this fraction exceeds a user-selectable fraction (default 0.5).

#### Extrapolate beyond image frame and time-interpolate occluded data (optional)

In addition to steps 1–4, two optional steps may be performed: one for facilitating extrapolation beyond the image frame, the other for time-interpolation of occluded data. Unlike steps 1–4 which are performed for each image, steps 5–6 can be performed after analyzing an entire image sequence. A video of TimTrack analysis may be used to decide whether to apply these optional steps (see S1 Video for example of gastrocnemius lateralis during jumping). If considerable extrapolation beyond the image frame occurred in the image sequence, the user may choose to enable step 5 (see S2 Appendix) and spread the extrapolation equally over left and right sides of the image (see S1 Fig). If image brightness dropped considerably during the image sequence (suggesting occlusion), the user may choose to enable step 6 (see S2 Appendix) and time-interpolate geometric features for images with brightness below a user-defined threshold (see S2 Fig). We decided not to apply steps 5 and 6 to our datasets, the former to facilitate comparison to manual observers, the latter because no substantial occlusion occurred. We do provide a video of TimTrack analysis on vastus lateralis during jumping, for which both extrapolation and time-interpolation were employed (see S2 Video).

### Experimental testing of algorithm

We employed the TimTrack algorithm on a variety of data, to test its functionality with a diversity of images and muscle geometries. We applied the algorithm to four different data sets, including a total of 26 different subjects, three different muscles, and four different ultrasound devices. We compared TimTrack’s estimates against manual estimates by three independent human observers for a subset of images within each data set (339 images in total). In addition, we compared TimTrack’s estimates to alternative algorithm estimates for a subset of data.

#### Dataset 1: Vastus Lateralis during isometric joint action

A sequence of vastus lateralis ultrasound images during isometric knee torque production in human subjects (6 male, 3 female). Images were obtained at rate of 30 Hz using a 5 cm ultrasound probe (11 MHz basic-mode; Logiq E9, General Electric, Fairfield, USA) as part of another study [25]. Prior to data collection, participants provided their written informed consent as approved by the University of Calgary Conjoint Health Research Ethics Board. We compared TimTrack’s estimates versus manual estimates for a subset of 153 images within this dataset.

#### Dataset 2: Gastrocnemius Lateralis during jumping and range-of-motion task

A sequence of gastrocnemius lateralis ultrasound images during (1) active ankle range-of-motion movement and (2) jumping in one human subject. Images were obtained at a rate of 25 Hz using a 5 cm ultrasound probe (11 MHz basic-mode; Logiq E9, General Electric, Fairfield, USA). Prior to data collection, the participant provided their written informed consent as approved by the University of Calgary Conjoint Health Research Ethics Board. We compared TimTrack’s estimates versus manual estimates for a subset of 56 images within this dataset.

#### Dataset 3: Gastrocnemius Medialis during isokinetic- and isometric joint action (by Drazan et al.)

A sequence of gastrocnemius medialis ultrasound images during various conditions in one human subject, collected by Drazan and colleagues [13] (see Data availability). These images were collected during (1) isokinetic ankle torque production at various velocities and (2) maximal isometric ankle torque production. Images were obtained at a rate of 60 Hz using a 6 cm ultrasound probe (LV7.5/60/128Z-2, SmartUs, TELEMED, Lithuania). We compared TimTrack’s estimates versus manual estimates for a subset of 100 images within this dataset. We also applied a commonly-used optic flow algorithm (i.e. UltraTrack) [12] on one trial of this dataset.

#### Dataset 4: Gastrocnemius Medialis images from different devices (by Seynnes & Cronin)

Individual gastrocnemius medialis ultrasound images in fifteen human subjects, collected by Seynnes and Cronin [15] (see Data availability). Two different probes/devices were used to collect the images: (1) a 6 cm probe (LV7.5/60/96Z, LogicScan 128 EXT-1Z, Telemed, Lithuania) and (2) a 5 cm probe (5–12 MHz HD11XE, Phillips, Bothell, Washington, USA). We compared TimTrack’s estimates on a subset of 30 images (i.e. ‘Sample A’ and ‘Sample B’, see [15]) against those from both the SMA algorithm created by Seynnes and Cronin [15], and human observers. The SMA algorithm is freely available as a macro tool in ImageJ software (NIH, Bethesda, MD, USA).

#### Parameter values and low-pass filtering

The algorithm was applied in similar manner to all four data sets, with a few minor changes in parameter and filter options. For step 0, only two parameters were adjusted, to indicate the aponeurosis locations (relative depth range *D*_superficial_ and *D*_deep_), and otherwise the same filtering parameters were used on all data (see S1 Appendix). For identification of aponeuroses, the default Hough transform method without aponeurosis filtering (see step 2) and with linear deep aponeurosis fit (*o*_deep_ = 1, see S1 Appendix) was used for data sets 1, 3 and 4 where the aponeuroses were relatively straight. In dataset 2, we found the aponeuroses to be curvilinear, and therefore selected the object detection method, with aponeurosis filtering (see step 2) and quadratic deep aponeurosis fit (*o*_deep_ = 2, see S1 Appendix). For fascicle tracking, we also adjusted the low-pass filter cut-off frequencies to reduce the effects of random noise. We used a cut-off of 1 Hz for slower movements (dataset 1, range-of-motion movement of dataset 2, 30 deg/s isokinetic movement of dataset 3) and 10 Hz for faster movements (all other data).

#### Outcome measures and statistics

The algorithm’s accuracy was determined from comparison to manual estimates from three independent observers, quantified with the root-mean-square difference (RMSD) and mean-absolute difference (MAD). The observers were biomechanics research staff members with a minimum of an undergraduate health science degree and at least one year of training and experience in ultrasonography and manual tracking. The observers performed manual tracking of images independent of the proposed algorithm, using ImageJ software (NIH, Bethesda, MD, USA). We treat their results as the standard for comparison, since there is no other generally accepted ground truth for fascicle tracking. To assess the algorithm’s (dimensionless) accuracy relative to current state-of-the-art algorithms, the coefficient of multiple correlations (CMC) [26] between the algorithm’s estimates and manual observer estimates was computed. To assess the variability within manual observer estimates, the difference between manual estimates of different observers was also quantified using the root-mean-square difference (RMSD) and the mean-absolute difference (MAD). The intraclass correlation coefficient (ICC) between manual observer estimates was computed to assess the difference in manual observer estimates relative to previously reported values. We consider ICC thresholds of 0.75 and 0.90 to indicate good and excellent reliability respectively, in line with [27]. Linear regressions were performed for muscle thickness, pennation angle and fascicle length with mean of manual estimate as independent variable and algorithm estimate as dependent variable. This was done to assess potential systematic error, which would be absent if the linear regression coefficient equals unity (1).

## Results

**Fig 3:**
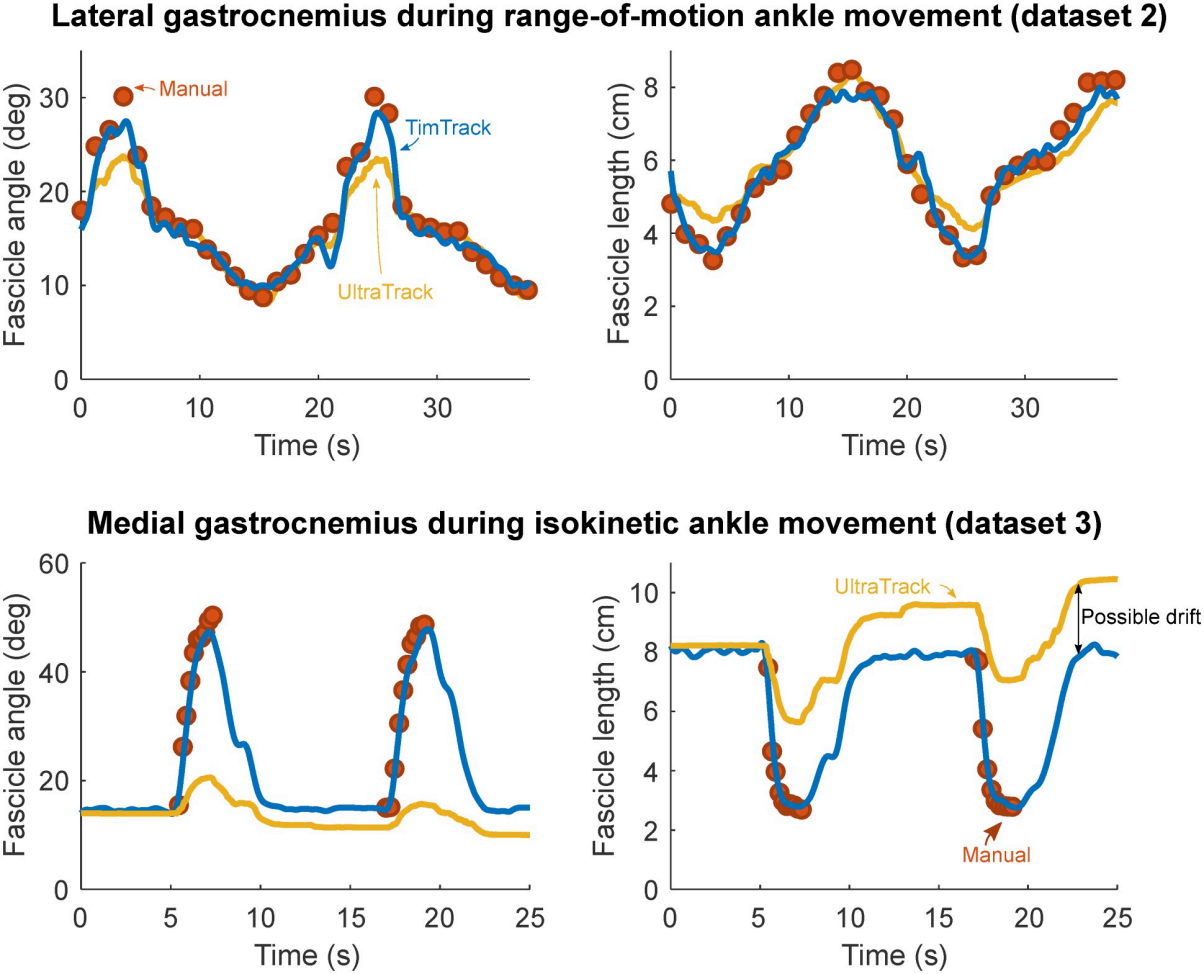
Fascicle angle and fascicle length estimates of TimTrack, UltraTrack and manual observers for two typical example trials. Fascicle angle and -length changed considerably during both movements, captured by manual observers (red dots), TimTrack (blue lines) and UltraTrack (yellow lines). Upper row: Two repetitions of a range-of-motion ankle movement from dataset 2. Lower row: Two repetitions of the 30 deg/s isokinetic ankle movement from dataset 3. UltraTrack overestimated fascicle length near the end of the trial, potentially due to integration drift. Manual estimation was performed on the concentric phase of each contraction, in accordance with the analysis by Drazan and colleagues [13].

**Fig 4:**
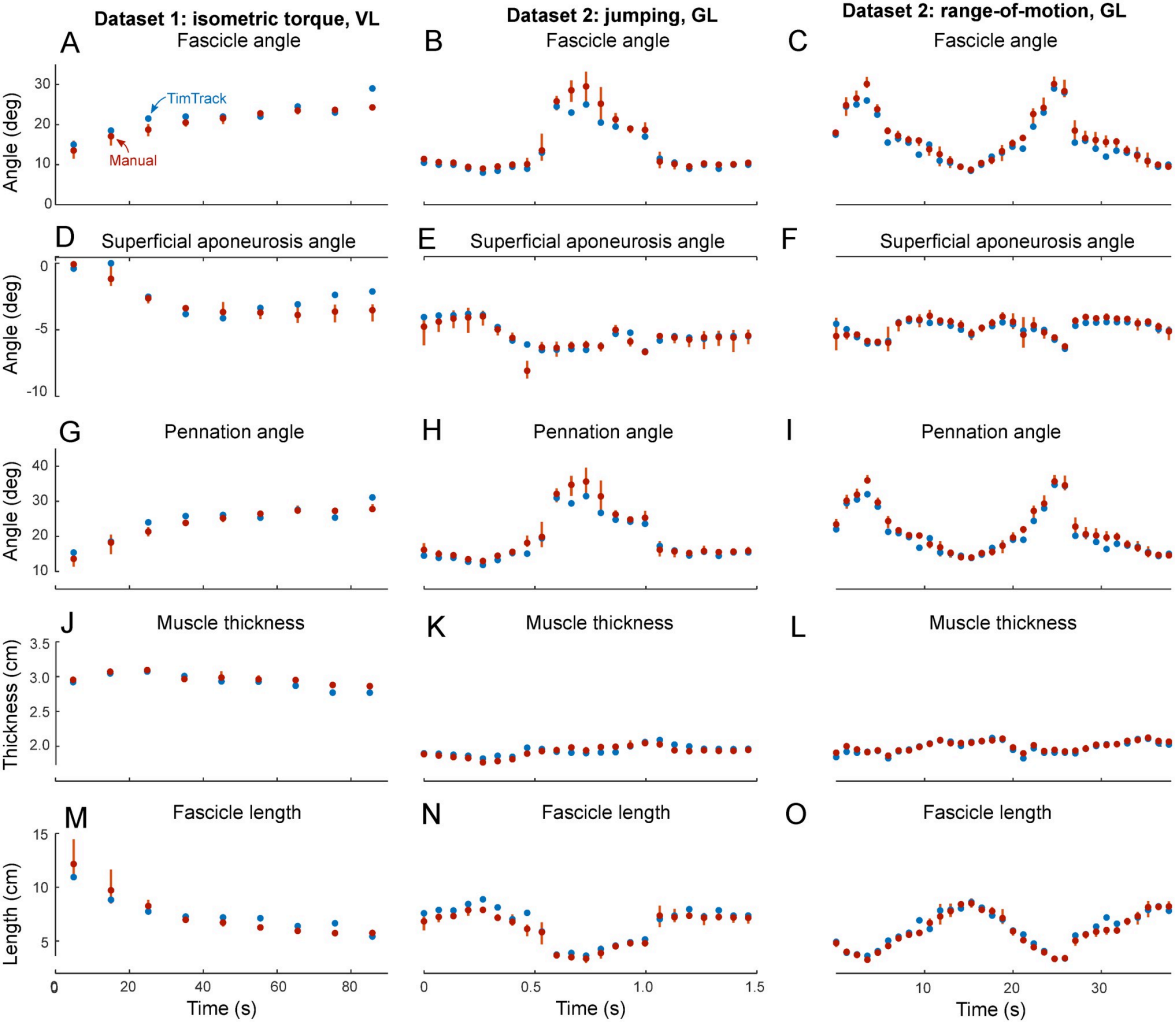
Estimates of TimTrack algorithm and manual observer on geometric muscle features in two datasets (1–2). Estimates of TimTrack algorithm (blue dots) and manual observers (red dots) for fascicle angle (A-C), superficial aponeurosis angle (D-F), pennation angle (G-I), muscle thickness (J-L) and fascicle length (M-O) of vastus lateralis (VL) during isometric torque production (left column), gastrocnemius lateralis (GL) during jumping (middle column) and gastrocnemius lateralis (GL) during range-of-motion movement (right column). Vertical red lines indicate the range of manual estimates for each image. When manual estimates are similar, this connecting line may not be visible. Similarly, when manual and TimTrack’s estimates are nearly identical, the latter may not be visible. Ultrasound images for manual estimation were selected such that they were spaced equidistantly and included the entire range of fascicle angles and -lengths present in each condition.

**Fig 5:**
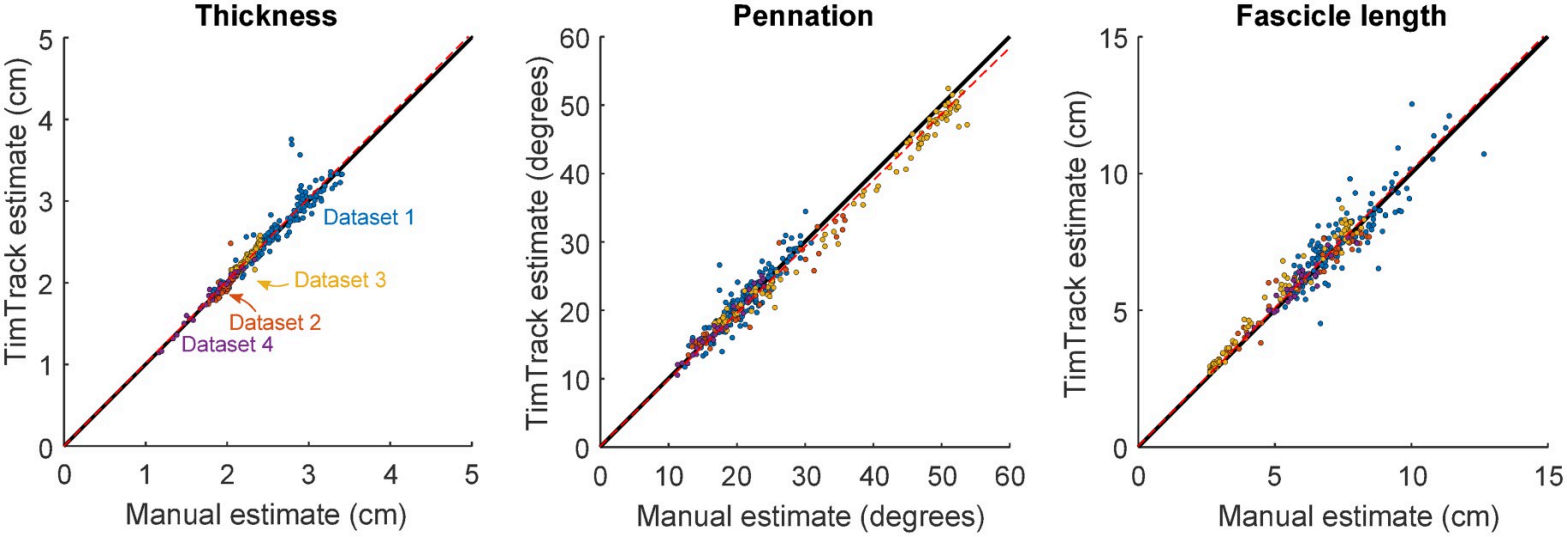
TimTrack algorithm estimates vs. mean manual observer estimates for all datasets. A. TimTrack algorithm estimates of muscle thickness increased linearly with mean of manual estimates (slope = 1.01±0.01, linear regression, dashed red line). B. TimTrack algorithm estimates of pennation angle increased linearly with mean of manual estimates (slope = 0.97±0.01, linear regression, dashed red line). C. TimTrack algorithm estimates of fascicle length increased linearly with mean of manual estimates (slope = 1.01±0.01, linear regression, dashed red line). Overall, TimTrack’s were closely related to mean observer estimates (R^2^ = 0.93–0.97).

The TimTrack algorithm estimated geometric muscle features relatively well in all four datasets. The algorithm provided accurate estimates of (changes in) fascicle length and fascicle angle (see Fig 3), as well as pennation angle and muscle thickness (see Fig 4). It performed well in comparison to manual tracking by human observers (see Tables 1–3), without the drift-sensitivity of the UltraTrack optic flow algorithm (see Fig 3), and with similar accuracy as the SMA algorithm. Estimation errors seemed to be mostly of random nature, with negligible under- or overestimation (see Fig 5).

**Table 1:** Muscle thickness.

	Manual-manual differences			Algorithm-manual differences		
	*MAD*	*RMSD*	*ICC*	*MAD*	*RMSD*	*CMC*
**Dataset 1**	0.04 cm	0.07 cm	0.99	0.08 cm	0.15 cm	0.96
**Dataset 2**	0.02 cm	0.02 cm	0.99	0.04 cm	0.07 cm	0.85
**Dataset 3**	0.03 cm	0.05 cm	0.96	0.05 cm	0.06 cm	0.94
**Dataset 4**	0.03 cm	0.05 cm	0.99	0.03 cm	0.04 cm	0.99
**Overall**	0.03 cm	0.06 cm	0.99	0.06 cm	0.11 cm	0.98

Mean-absolute difference (MAD), root-mean-square difference (RMSD) and intraclass correlation coefficient (ICC) between manual observer estimates, as well as MAD, RMSD and correlation of multiple coefficients (CMC) between manual observer estimates and TimTrack algorithm estimates.

**Table 2:** Pennation angle.

	Manual-manual differences			Algorithm-manual differences		
	*MAD*	*RMSD*	*ICC*	*MAD*	*RMSD*	*CMC*
**Dataset 1**	1.70 deg	2.14 deg	0.94	1.23 deg	1.70 deg	0.96
**Dataset 2**	1.34 deg	1.77 deg	0.99	1.20 deg	1.77 deg	0.98
**Dataset 3**	1.19 deg	1.60 deg	0.99	1.77 deg	2.21 deg	0.99
**Dataset 4**	0.74 deg	0.88 deg	0.99	0.67 deg	0.82 deg	0.99
**Overall**	1.39 deg	1.83 deg	0.99	1.34 deg	1.82 deg	0.99

Mean-absolute difference (MAD), root-mean-square difference (RMSD) and intraclass correlation coefficient (ICC) between manual observer estimates, as well as MAD, RMSD and correlation of multiple coefficients (CMC) between manual observer estimates and TimTrack algorithm estimates.

**Table 3:** Fascicle length.

	Manual-manual differences			Algorithm-manual differences		
	*MAD*	*RMSD*	*ICC*	*MAD*	*RMSD*	*CMC*
**Dataset 1**	0.58 cm	0.75 cm	0.93	0.46 cm	0.66 cm	0.94
**Dataset 2**	0.41 cm	0.55 cm	0.97	0.28 cm	0.38 cm	0.98
**Dataset 3**	0.18 cm	0.26 cm	0.99	0.27 cm	0.37 cm	0.99
**Dataset 4**	0.22 cm	0.28 cm	0.98	0.23 cm	0.30 cm	0.98
**Overall**	0.39 cm	0.56 cm	0.98	0.36 cm	0.52 cm	0.98

Mean-absolute difference (MAD), root-mean-square difference (RMSD) and intraclass correlation coefficient (ICC) between manual observer estimates, as well as MAD, RMSD and correlation of multiple coefficients (CMC) between manual observer estimates and TimTrack algorithm estimates.

TimTrack’s estimates generally agreed well with those from manual observers, without the drift-sensitivity of optic flow algorithms. As an example, we compared TimTrack and UltraTrack for two typical example trials (see Fig 3), one from dataset 2 without much drift (upper row) and another from dataset 3 with some apparent drift towards the end of the sequence (lower row). Apart from drift insensitivity, TimTrack seemed better able to capture the relatively large fascicle angles and small fascicle lengths in both trials. Overall, TimTrack appeared to perform well in comparison with mean of manual estimates for these trials, without drift sensitivity.

TimTrack algorithm estimates were generally comparable to manual estimates, as shown for three typical-example trials (see Fig 4). Algorithm-manual RMSDs were comparable to manual-manual RMSDs (see Tables 1–3), indicating that the algorithm’s estimation was like that of the human observers. Overall, TimTrack’s RMSDs for muscle thickness, pennation angle and fascicle length were 0.11 cm, 1.82 deg and 0.52 cm respectively (see Tables 1–3), comparable to the corresponding RMSDs between manual observers (i.e., 0.06 cm, 1.83 deg and 0.56 cm respectively, see Tables 1–3). For dataset 1, the algorithm provided relatively accurate estimates for all nine subjects, with a fascicle length RMSD of 0.63 ± 0.20 cm (mean ± s.d.). For dataset 2, the algorithm performed well for both a slow, range-of-motion movement (fascicle length RMSD = 0.36 cm) and a fast, jumping movement (fascicle length RMSD = 0.41 cm, see S1 Video). For dataset 3, TimTrack provided accurate estimates across a large variety of conditions, including various isokinetic dynamometer speeds. The algorithm performed well in both the fastest isokinetic condition (i.e. 500 deg/s, fascicle length RMSD = 0.48 cm) and the slowest isokinetic condition (i.e. 30 deg/s, RMSD = 0.30 cm). For dataset 4, TimTrack performed well for images acquired with both Phillips and Telemed systems, which varied in image brightness, resolution, and speckle. TimTrack’s fascicle length RMSDs for images acquired with these two systems were 0.35 cm and 0.23 cm respectively, comparable to those from both the SMA algorithm (0.47 cm and 0.30 cm) and human observers (0.32 cm and 0.23 cm). Overall, TimTrack provided accurate estimates of geometric muscle features for ultrasound images acquired from various human subjects for a broad range of movement conditions, with relatively little sensitivity to image brightness, resolution, and speckle.

The algorithm’s estimate of muscle thickness, pennation angle and fascicle length increased with (the mean of the) observer estimate (slope = 1.01 ± 0.01, 0.97 ± 0.01 and 1.01 ± 0.01 respectively, estimate ± 95% confidence interval, linear regression, see Fig 5). In addition to all regression slopes being close to 1, the algorithm vs. manual estimates of thickness, pennation and fascicle length were well explained by the identity lines (R^2^ = 0.94, R^2^ = 0.97 and R^2^ = 0.93, respectively), indicating that there was negligible systematic under- or overestimation.

## Discussion

The TimTrack algorithm estimates geometric muscle features from ultrasound images, including muscle thickness, superficial aponeurosis angle, fascicle angle, fascicle length and pennation angle. We found reasonably good agreement between algorithm- and manual estimates for human gastrocnemius medialis- and lateralis, and vastus lateralis muscle, with algorithm-manual differences comparable to manual-manual differences. This indicates that TimTrack may be suitable for replacing manual estimation in experimental studies where these geometric muscle features are of interest. In the following, we compare TimTrack to other contemporary algorithms, considering the respective assumptions, limitations, and performance.

TimTrack provides a high level of automation while employing state-of-the-art features from several other algorithms. We found good accuracy to be achieved with the use of Frangi-type Hessian filtering in combination with Hough transform for fascicle detection, similar to employed previously [19]. We combined this technique with automatic aponeurosis detection for muscle thickness estimation, adopting either object detection, or Hough transform. These detection procedures were not highly sensitive to hyper-parameter selection. This was demonstrated by using the same parameter set for analysis of three different muscles in a variety of human subjects and movement conditions, including some images captured with entirely different ultrasonography systems, differing in image brightness, resolution, and speckle. The combination of filtering line-like structures and line-detection procedures in TimTrack is similar to the algorithms from Zhou and colleagues [14, 20, 21] and the SMA algorithm from Seynnes and Cronin [15]. The former algorithms typically use Gabor wavelet filtering instead of vessel enhancement and Radon transform instead of Hough transform for aponeurosis detection. Vessel enhancement filtering has been found to be faster (about 17 times) than Gabor filtering, while yielding similar accuracy in muscle thickness estimation [14]. Radon- and Hough transform are closely related, the latter being faster in most cases [28]. Another difference is that algorithms from Zhou and colleagues typically select the most dominant fascicle angle from the Hough or Radon transform, while TimTrack uses the weighted median of several dominant angles. We prefer the latter because we found it to yield better results for images with non-uniform fascicle orientations, when the dominant orientation does not necessarily reflect the overall fascicle angle. In line with this observation, we found overall fascicle length RMSD to be 63% larger when only using the most dominant orientation instead of our weighted-median approach. Of these two alternative feature detection algorithms (i.e., Zhou and SMA), SMA is freely available for direct comparison. SMA also employs the Frangi-type vessel enhancement filter, amongst several other filter techniques. More extensive filtering may in part explain why SMA requires about 12 times more time per image than TimTrack (9.74 s vs. 0.81 s, Intel(R) Core i5-8250U CPU, 1.60 GHz). Despite extensive filtering, SMA achieved similar accuracy as TimTrack, as indicated by comparable fascicle length RMSD with manual estimates (0.40 cm vs. 0.30 cm). Altogether, the proposed TimTrack algorithm employs several approaches to improve objectivity, reproducibility and time efficiency, while achieving similar accuracy as alternative algorithms.

TimTrack is fundamentally different from optic flow-based algorithms [11–13] as its estimates do not depend on a succession of ultrasound images. The main advantage of optic flow-based algorithms is that they are relatively insensitive to speckle, and therefore have low requirements on image quality. This is because optic flow only detects global movement, while local speckle is effectively filtered out. Their main disadvantage is sensitivity to integration drift, which is usually manually corrected for longer image sequences, for example the images examined here (see Fig 3). For these images, TimTrack agrees well with human observers compared to the state-of-the-art UltraTrack optic flow algorithm. This was accomplished with minimal manual intervention, to set the regions of interest for aponeurosis detection, and without need for manual drift correction or initial fascicle identification. TimTrack’s estimates were also of similar accuracy as those by a more recent point tracking optic flow algorithm by Drazan and colleagues, which has been found to be more accurate than UltraTrack [13]. We employed TimTrack on a typical example subject from the Drazan study [13] (i.e. our dataset 3), resulting in fascicle length and pennation angle RMSDs of 0.37 cm and 2.21 deg (see Tables 2 and 3). In comparison, their point tracking algorithm yielded RMSDs of 0.59 cm and 7.16 deg when in ‘unsupervised’ mode and of 0.33 cm and 4.12 deg when in ‘supervised’ mode, on their overall dataset. TimTrack is more automated than their ‘supervised’ mode, while similarly accurate. TimTrack is similarly automated as their ‘unsupervised’ mode, while more accurate. Overall, TimTrack can achieve comparable accuracy to optic flow methods, while having some advantages with respect to drift and manual corrections.

There are nonetheless limitations and sensitivities to TimTrack. For example, image speckle may inadvertently cause a sub-population of fascicles within an image to dominate the calculations, and can thus affect the estimated fascicle orientation from the Hough transform. TimTrack therefore should work best on ultrasound images with relatively high quality and little speckle. Although TimTrack allows for curved aponeuroses, it presently includes only relatively simple, polynomial fits. It also assumes fascicles to be linear, similar to the other algorithms discussed here. It is therefore challenging to estimate lengths of curvilinear fascicles, especially when there is substantial speckle. The application of automated methods, including ours, should therefore be limited to movements where fascicles are fairly linear. Curvature and irregular fascicle patterns also provide a challenge to manual procedures, especially when extrapolation is required. For both automated and manual estimates, it is important to guard against errors due to such effects when extrapolating beyond the image frame.

## Conclusion

We here present an automated ultrasound algorithm that provides estimates of geometric muscle features without drift sensitivity of optic flow algorithms, and with more automation than most previous algorithms. Automation allows for relatively fast and objective analysis of many images or image sequences, while retaining accuracy comparable to manual estimations. These features may prove advantageous for analyzing cyclic movements such as locomotion, or movements with long image sequences. The TimTrack algorithm estimates geometric muscle features with good accuracy, as tested under force conditions comparable to locomotion.

## Supporting information

S1 Fig Extrapolation of muscle fascicle beyond image frame. The fascicle of interest (thick red line) goes through the midpoint *M*, and intersects with aponeuroses at locations outside of the image frame. The horizontal coordinate of the midpoint *M* is halfway the image width *w*. The vertical coordinate of the midpoint *M* is halfway between the deep and superficial aponeuroses. The selected width is determined by finding the intersection between the extrapolated fascicle of interested and the extrapolated deep aponeurosis.
(TIF)

S2 Fig Information to help decide on whether to use extrapolation mode and/or time-interpolation. Data corresponds to sequence of vastus lateralis ultrasound images during jumping (see S2 Video). Left: fraction of the fascicle extrapolated beyond the image frame (0–1). If a considerable portion is above a user-defined threshold value (default 0.5), we recommend using ‘extrapolate mode’ to equally spread extrapolation over left and right sides of the image. Right: image brightness, defined as the average grayscale value (0–1), i.e., averaged across all pixels within each image. We recommend using time-interpolation for images with brightness below a user-defined threshold (default 50% of time-average).
(TIF)

S1 Video TimTrack analysis of gastrocnemius lateralis muscle during jumping movement. Algorithm steps 1–4 were employed with object detection aponeurosis method and quadratic deep aponeurosis fit.
(GIF)

S2 Video TimTrack analysis of vastus lateralis muscle during jumping movement. Algorithm steps 1–6 were employed with Hough transform aponeurosis detection method and linear deep aponeurosis fit, and including optional steps for extrapolation (step 5) and time-interpolation (step 6).
(GIF)

S1 Appendix Parameter values. Description of parameters and list of parameter values for datasets 1–4.
(PDF)

S2 Appendix Optional steps in the algorithm. Optional extrapolation and time-interpolation steps.
(PDF)
