# Supplementary figures and images for: TimTrack: A drift-free algorithm for estimating geometric muscle features from ultrasound images
Source: PLoS One. 2022 Mar 24;17(3):e0265752. doi: 10.1371/journal.pone.0265752 (PMC8947026; doi:10.1371/journal.pone.0265752)

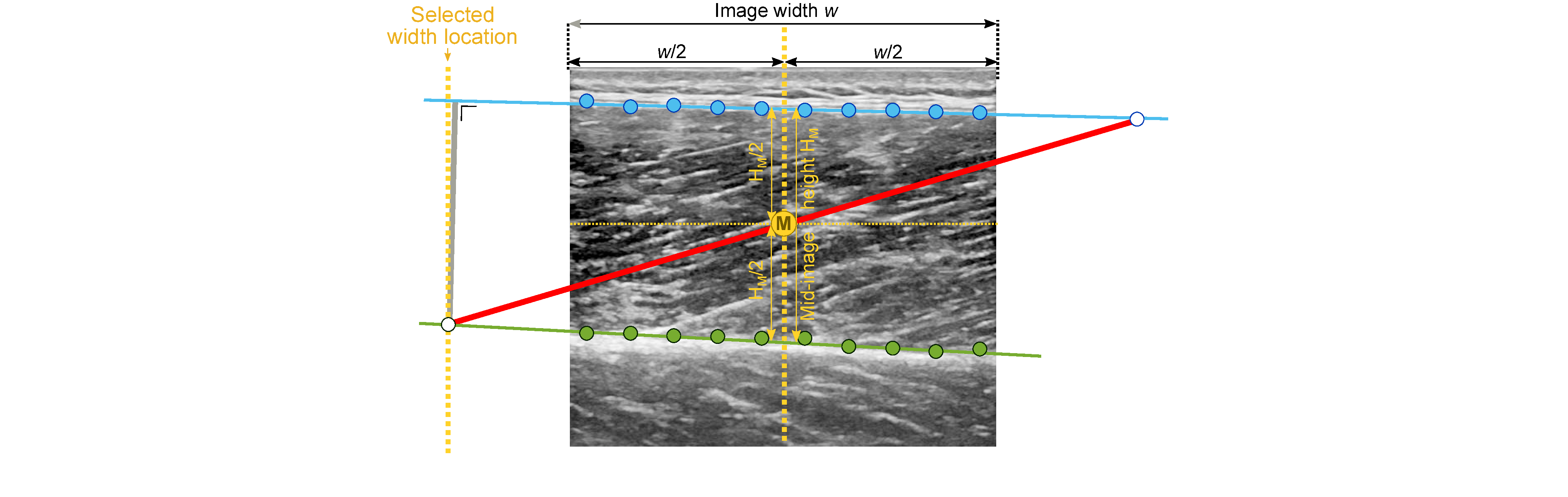

Supplement: S1 Fig — The fascicle of interest (thick red line) goes through the midpoint M, and intersects with aponeuroses at locations outside of the image frame. The horizontal coordinate of the midpoint M is halfway the image width w. The vertical coordinate of the midpoint M is halfway between the deep and superficial aponeuroses. The selected width is determined by finding the intersection between the extrapolated fascicle of interested and the extrapolated deep aponeurosis. (TIF) [file pone.0265752.s001.tif]

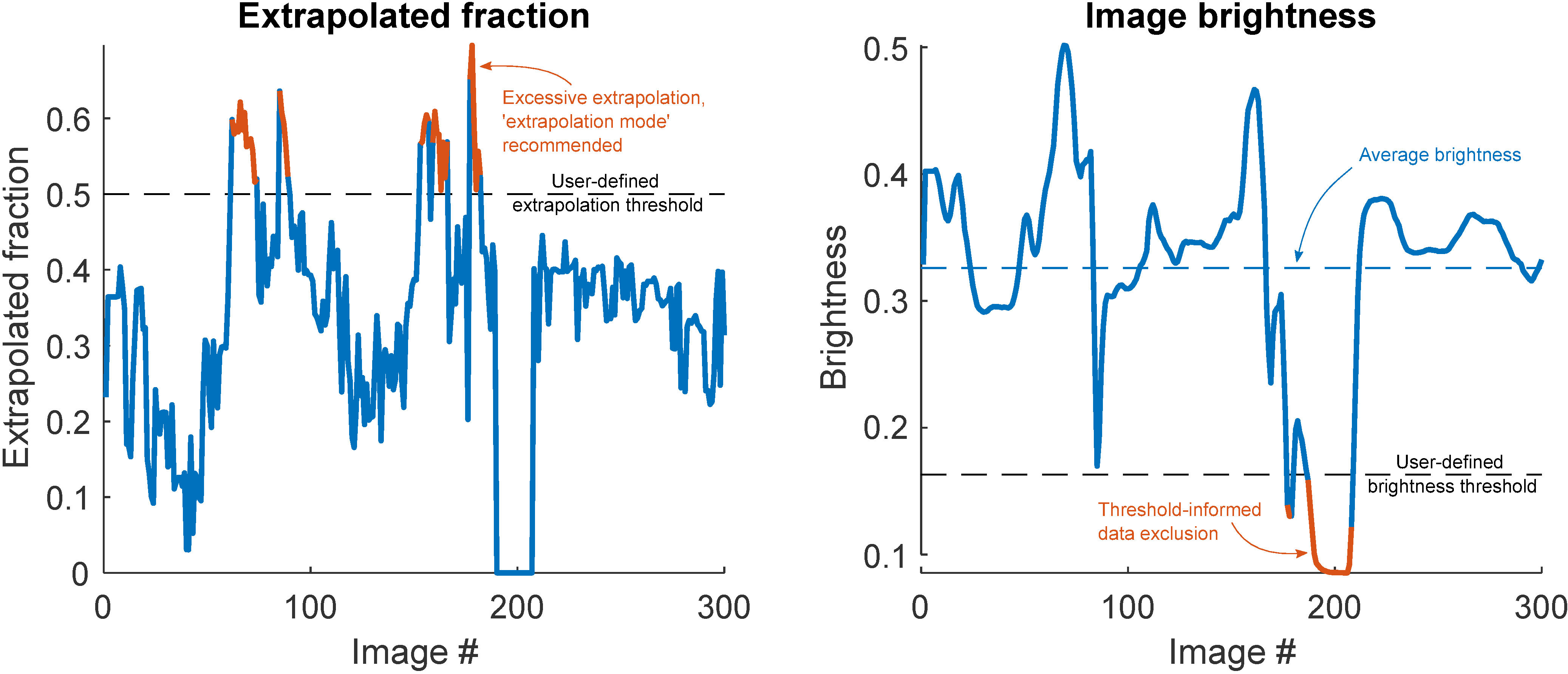

Supplement: S2 Fig — Data corresponds to sequence of vastus lateralis ultrasound images during jumping (see S2 Video). Left: fraction of the fascicle extrapolated beyond the image frame (0–1). If a considerable portion is above a user-defined threshold value (default 0.5), we recommend using ‘extrapolate mode’ to equally spread extrapolation over left and right sides of the image. Right: image brightness, defined as the average grayscale value (0–1), i.e., averaged across all pixels within each image. We recommend using time-interpolation for images with brightness below a user-defined threshold (default 50% of time-average). (TIF) [file pone.0265752.s002.tif]

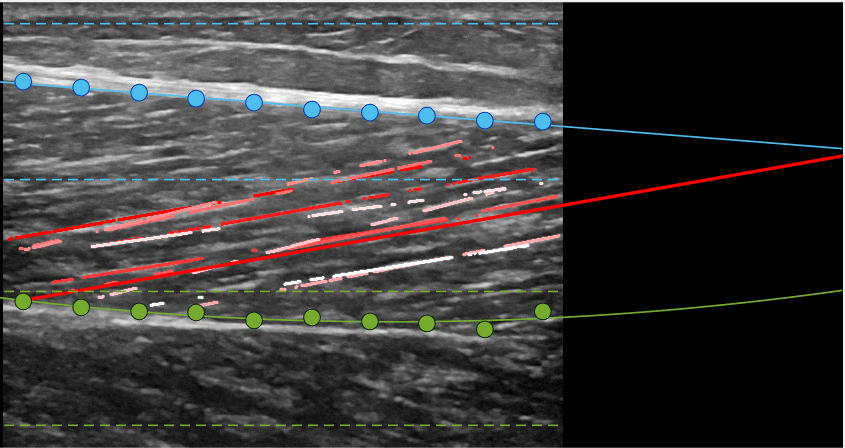

Supplement: S1 Video — Algorithm steps 1–4 were employed with object detection aponeurosis method and quadratic deep aponeurosis fit. (GIF) [file pone.0265752.s003.gif]

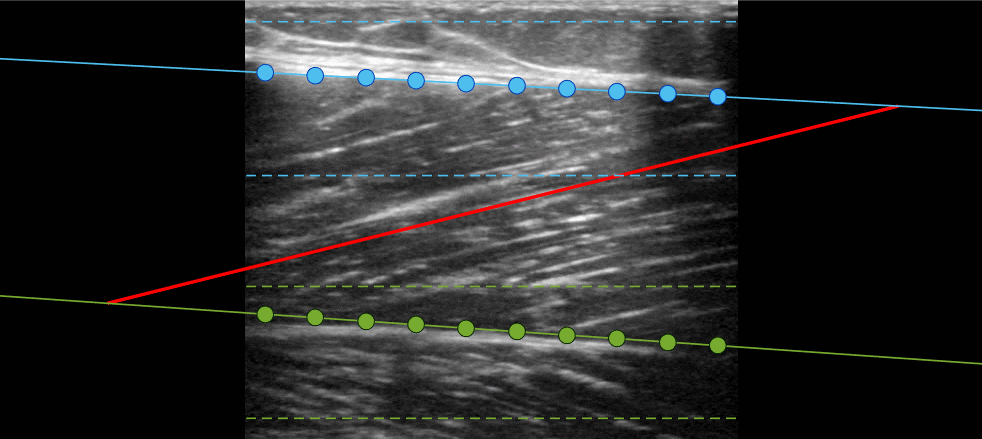

Supplement: S2 Video — Algorithm steps 1–6 were employed with Hough transform aponeurosis detection method and linear deep aponeurosis fit, and including optional steps for extrapolation (step 5) and time-interpolation (step 6). (GIF) [file pone.0265752.s004.gif]
