# Supplementary material for: TimTrack: A drift-free algorithm for estimating geometric muscle features from ultrasound images
Source: PLoS One. 2022 Mar 24;17(3):e0265752. doi: 10.1371/journal.pone.0265752 (PMC8947026; doi:10.1371/journal.pone.0265752)
Supplement: S1 Appendix — Description of parameters and list of parameter values for datasets 1–4. (PDF) [file pone.0265752.s005.pdf]

S1 Appendix: Parameter values

Parameter values

The parameters and their values for the current datasets are listed in the tables below. When using the algorithm on similar ultrasound images, most parameter values may be left unchanged. However, for considerably different images, parameter changes may be required. We provide a MATLAB-based software tool to easily change parameters, which is provided in the repository (see Data availability). This tool allows for changing parameters on a typical example image and observing their effect right away.

Parameters

The region selection parameters $D_{\mathrm{superficial}}$ and D_{deep} relate to the depth of the image, as some depth settings may cause the aponeuroses to reside outside the upper and lower halves of the image, respectively. The filtering parameters relate to the brightness and resolution of the collected image. For example, line thickness parameters for fascicles and aponeuroses (σ_{fas} and σ_{apo}) relate to the (average) thickness of these structures in terms of pixels, which depends on image resolution. Threshold parameters for fascicles and aponeuroses (T_{fas} and T_{apo}) relate to the brightness of these structures, which depends on the overall brightness of the image. Parameters for aponeurosis detection and fitting, and fascicle angle estimation relate to muscle properties and to image quality. For example, some muscles may have aponeurosis and fascicle angles that are outside the range used here (β_{range}, γ_{range} and $\theta_{\mathrm{fas,range}}$). The amount of fascicle angles selected in Hough transform (K) may depend on image quality, as fewer lines may be available in images with poorer quality.

Step 1: Filtering			
σ_{fas}	Fascicle thickness	1-2 pixels	1.1f
σ_{apo}	Aponeurosis thickness	18-20 pixels	1.1a & 1.4a
T_{fas}	Brightness threshold for fascicles	50% brightest	1.2f
T_{apo}	Brightness threshold for aponeurosis	50% brightest	1.2a & 1.5a
Step 2: Aponeurosis point detection and fitting			
$\theta_{\text{apo,res}}$	Angle resolution in fascicle Hough transform	0.5°	2.1 – Hough
$L_{\text{ratio,max}}$	Maximum length ratio between longest and second-longest aponeurosis objects	0.9	2.1 – object detection
x_{margin}	Margin of horizontal aponeurosis points	20 pixels	2.1
n_{apox}	Number of points in aponeurosis points	10	2.1
o_{super}	Order of the super aponeurosis fit	1	2.2
β_{range}	Range of eligible superficial aponeurosis angles β	-45° to +1°	2.2
γ_{range}	Range of eligible deep aponeurosis angles γ	-45° to +1°	2.2
Step 3: Fascicle angle estimation			
$\theta_{\text{fas,range}}$	Range of eligible fascicle angles	8° to 80°	3
$\theta_{\text{fas,res}}$	Angle resolution in fascicle Hough transform	0.5°	3
K	Amount of selected fascicle Hough peaks	10	3

Parameter values applied to all datasets

Parameter	Description	Dataset 1	Dataset 2	Dataset 3	Dataset 4
$D_{\text{superficial}}$	Superficial aponeurosis depth range	0.05-0.40	0.05-0.40	0.02-0.20	0.03-0.30
D_{deep}	Deep aponeurosis depth range	0.60-0.95	0.60-0.95	0.30-0.70	0.30-0.90
o_{deep}	Order of deep aponeurosis fit	1	2	1	1

Dataset-specific depth range- and aponeurosis fit parameter values
