# Supplementary material for: TimTrack: A drift-free algorithm for estimating geometric muscle features from ultrasound images
Source: PLoS One. 2022 Mar 24;17(3):e0265752. doi: 10.1371/journal.pone.0265752 (PMC8947026; doi:10.1371/journal.pone.0265752)
Supplement: S2 Appendix — Optional extrapolation and time-interpolation steps. (PDF) [file pone.0265752.s006.pdf]

S2 Appendix: Optional steps in the algorithm

After analyzing an image sequence, the user may choose to perform TimTrack's optional steps, which facilitate extrapolation beyond the image frame and time-interpolating for occluded images. To help the user decide, TimTrack can show the extrapolated fraction of the fascicle and the average image brightness.

Optional step 5: Extrapolate geometry beyond the image frame for longer fascicles

If the muscle fascicles extend beyond the width of the image, the algorithm can facilitate extrapolation of aponeuroses and fascicles using the 'extrapolate mode' (see S1 Fig). In extrapolation mode, the selected horizontal location x for each image is chosen such that the extrapolation is spread between left and right sides of the image. This is accomplished by requiring the fascicle of interest to go through the midpoint M of the image (see S1 Fig). The selected horizontal location x is then defined as the horizontal coordinate of the intersection between the extrapolated deep aponeurosis and the extrapolated fascicle of interest. The 'extrapolation mode' is demonstrated here with the vastus lateralis, which had relatively long fascicles extending beyond the image frame (see S1 Fig). While 'extrapolation mode' facilitates extrapolation through equal division between left and right sides of the image, it should only be employed when the user deems it appropriate. To decrease the risk of erroneous extrapolation, the algorithm can optionally create a video of the analysis, allowing the user to review the extrapolation (see S2 Video). If extrapolation on one side of the image exceeds 50% of the image width, TimTrack warns the user that estimates may be inaccurate. To help the user decide whether to use 'extrapolation mode', TimTrack can optionally show the extrapolated fraction of the fascicle for each image in the sequence (see S2 Fig).

Optional step 6: Time-interpolate aponeuroses for image sequences with missing data

Ultrasound images may be temporarily occluded when the ultrasound probe loses contact with the skin, for example during high-acceleration movements such as landing a jump. In these cases, aponeuroses and fascicles may only be partially visible, and their estimated positions may be inaccurate. If the user suspects that this occurs, TimTrack can identify occluded images using an image brightness criterion, and replace the corresponding estimates using time-interpolation. To decrease the risk of erroneous interpolation, the algorithm allows the user to review a recorded video of the analysis (see S2 Video). While the post-hoc time interpolation option can be useful when images are temporarily occluded, whether to perform interpolation is decided by the user. To further help the user with their decision, TimTrack can optionally show the average image brightness for each image in the sequence (see S2 Fig).
